# Strain Sensor-Based Fatigue Prediction for Hydraulic Turbine Governor Servomotor in Complementary Energy Systems

**DOI:** 10.3390/s25185860

**Published:** 2025-09-19

**Authors:** Hong Hua, Zhizhong Zhang, Xiaobing Liu, Wanquan Deng

**Affiliations:** 1 Key Laboratory of Fluid and Power Machinery, Ministry of Education, Xihua University, Chengdu 610039, China; 2 CHN Energy Dadu River Zhentouba Power Generation Co., Ltd., Leshan 614900, China

**Keywords:** strain sensor, hydraulic turbine governor, servomotor, fatigue prediction, finite element analysis

## Abstract

Hydraulic turbine governor servomotors in wind solar hydro complementary energy systems face significant fatigue failure challenges due to high-frequency regulation. This study develops an intelligent fatigue monitoring and prediction system based on strain sensors, specifically designed for the frequent regulation requirements of complementary systems. A multi-point monitoring network was constructed using resistive strain sensors, integrated with temperature and vibration sensors for multimodal data fusion. Field validation was conducted at an 18.56 MW hydroelectric unit, covering guide vane opening ranges from 13% to 63%, with system response time <1 ms and a signal-to-noise ratio of 65 dB. A simulation model combining sensor measurements with finite element simulation was established through fine-mesh modeling to identify critical fatigue locations. The finite element analysis results show excellent agreement with experimental measurements (error < 8%), validating the simulation model approach. The fork head was identified as the critical component with a stress concentration factor of 3.4, maximum stress of 51.7 MPa, and predicted fatigue life of 1.2 × 10^6^ cycles (12–16 years). The cylindrical pin shows a maximum shear stress of 36.1 MPa, with fatigue life of 3.8 × 10^6^ cycles (16–20 years). Monte Carlo reliability analysis indicates a system reliability of 51.2% over 20 years. This work provides an effective technical solution for the predictive maintenance and digital operation of wind solar hydro complementary systems.

## 1. Introduction

Under the global energy transformation context, wind solar hydro complementary power generation systems have received widespread attention due to their complementary characteristics and regulation capabilities [1]. These systems utilize hydroelectric regulation to address the intermittency of wind and photovoltaic generation, improving grid frequency stability and power balance capabilities [2]. Research indicates that the complementary characteristics of wind and hydro power can significantly improve the risk profile of energy inflows and reduce system operational uncertainty [3]. However, this integration fundamentally transforms hydraulic turbine governing systems from steady-state operators into dynamic regulation providers, creating unprecedented operational demands that subject governor actuators, particularly guide vane servomotors, to complex cyclic loading patterns far exceeding traditional design assumptions [4].

As the core regulation device of complementary energy systems, hydraulic turbine governing systems must frequently respond to renewable energy fluctuations, with regulation frequencies potentially reaching 8760 cycles annually compared to 2000–3000 cycles in conventional hydropower plants. This high-frequency regulation mode subjects governor actuators, particularly guide vane servomotors, to numerous cyclic loads, significantly increasing fatigue failure risk [4]. Fatigue failure has become one of the most common failure modes in hydraulic turbine equipment, primarily caused by the combined effects of stress concentration, material defects, and cyclic loading [5]. Machine learning technology shows tremendous potential in hydroelectric equipment fatigue optimization, with recent advances demonstrating sophisticated approaches to complex loading scenarios. Active learning-based optimization methods can effectively reduce fatigue damage during turbine startup processes [6], with machine learning algorithms capable of reducing fatigue damage by up to 50% [7]. Hybrid methodologies combining experimental data and machine learning techniques provide new approaches for evaluating hydroelectric units under flexible operating conditions [8]. However, these data-driven approaches often require extensive training datasets from similar operational conditions, which are frequently unavailable for the relatively novel complementary energy system configurations, limiting their applicability to high-frequency regulation scenarios.

Sensor technology development provides powerful tools for hydraulic machinery health monitoring, with multi-sensor systems demonstrating exceptional capabilities for detecting various operational anomalies and fatigue-related phenomena. Research on different sensor types for detecting hydraulic phenomena in Francis turbines shows that multi-sensor systems can effectively identify various operational anomalies [9]. Flow-induced fatigue damage has become a major threat to large Francis turbines under multiple operational loads [10], with complex flow patterns in complementary systems creating additional challenges for traditional monitoring approaches. Rainflow counting matrix interpolation methods show good application prospects in fatigue assessment under different operating conditions [7]. Advanced strain sensor technologies have demonstrated remarkable performance in challenging industrial environments, providing the foundation for sophisticated structural health monitoring applications. Hydraulic turbine head cover fastening bolt deformation monitoring systems provide important safeguards for large hydroelectric equipment safe operation [11]. Successful applications of strain virtual sensing technology in industrial press fatigue monitoring [12] and the excellent fatigue performance of Type I fiber Bragg grating strain sensors [13] provide technical references for hydraulic turbine fatigue monitoring. Despite these technological advances, most existing sensor applications focus on primary turbine components such as runners and main shafts, with limited attention to the critical governor actuator components that experience severe loading in complementary energy systems.

Comprehensive fatigue strength analysis methodologies have been developed for prototype Francis turbines, establishing systematic procedures from instrumentation design to field measurement validation. Prototype Francis turbine fatigue strength analysis research establishes complete assessment procedures from instrumentation to field measurements [14], while the multi-axial fatigue analysis of 100 MW hydro-generator unit shafting provides important experience for large equipment fatigue assessment [15]. Advanced signal processing techniques have shown promise for predicting turbine component behavior under complex operational conditions. Hydraulic turbine runner strain signal prediction technology achieves high-precision prediction through cyclostationary decomposition and Kriging interpolation methods [16], though these methods require validation for the variable loading patterns characteristic of complementary energy systems. Research on load spectrum assumption effects on turbine reliability assessment reveals the importance of accurate load modeling [17]. Simulation model technology is increasingly applied in hydraulic turbine monitoring, offering sophisticated approaches for real-time analysis and predictive maintenance. Virtual sensor implementation in turbines provides simulation model methods for gap violation analysis [18], while strain sensor applications in turbine vortex detection and visualization show good prospects [19]. Numerical research methods for multilevel lifetime assessment procedures provide systematic solutions for prototype Francis turbine fatigue strength analysis [20]. However, current digital modeling implementations often focus on primary turbine components, with simplified representations of governor mechanisms that may not capture the complex stress concentrations and contact phenomena in servomotor assemblies under high-frequency operation.

Fatigue reliability research in renewable energy systems provides valuable insights that can be adapted for hydraulic turbine applications, particularly in understanding the effects of variable-amplitude loading on component durability. Posterior stress range prediction based on strain measurements in wind turbine support structure fatigue reliability analysis achieves good results [21]. Load matching complementarity characteristics research has revealed the complex interaction patterns between different renewable energy sources and their implications for hydroelectric regulation requirements. Research on load matching complementarity characteristics in wind solar complementary systems shows that reasonable system configuration can significantly improve overall operational efficiency [22]. The dynamic modeling and stability analysis of hydraulic turbine governing systems has become increasingly important as system complexity grows with complementary energy integration. Dynamic modeling and stability analysis of hydraulic turbine governing systems has become a research focus [23]. Experimental investigations have provided critical insights into fatigue damage mechanisms, though most studies focus on startup and shutdown events rather than continuous high-frequency regulation. Strain measurement-based fatigue analysis of prototype Francis turbines provides important references for practical engineering applications [24], while experimental research on fatigue damage during hydroelectric unit startup processes reveals key damage mechanisms [25]. Advanced monitoring technologies for wind energy systems offer insights that could be adapted for hydraulic turbine applications. Machine learning approaches show promise for wind turbine efficiency optimization through smart failure detection and strategic component placement [26]. Comprehensive fatigue assessment methodologies have been developed for wind turbine systems, providing frameworks that could be adapted for hydraulic applications. Fatigue assessment of wind turbine towers demonstrates the value of strategic processing approaches with illustrative case studies [27]. Sensor integration technologies for drivetrain testing show potential for adaptation to hydraulic turbine monitoring applications. From strain measurements to load determination, the development of measurement solutions for wind turbine transmission input loads during drivetrain testing provides valuable methodological insights [28].

Sediment erosion effects on turbine components represent an additional factor that must be considered in comprehensive fatigue analysis, particularly for installations in sediment-laden watersheds. Fatigue damage assessment of turbine runner blades considering sediment wear effects provides important insights into multi-factor degradation mechanisms [29]. Comprehensive regulation benefits analysis has demonstrated the value of hydropower in reducing renewable energy fluctuations, though the mechanical implications for regulation equipment require further investigation. The comprehensive regulation benefits of hydropower generation systems in reducing wind power fluctuation have been well documented [2]. Multi-energy complementary system optimization across multiple temporal scales reveals the complexity of the operational demands placed on regulation equipment. Research exploring the sensitivity of capacity configuration for multi-energy complementary systems across multi-temporal scales provides system-level context [30]. Vibration avoidance strategies in multi-objective optimization demonstrate the importance of considering multiple performance criteria in complementary system design. The multi-objective optimization of hydro-wind-photovoltaic power complementary plants with vibration avoidance strategies shows the complexity of system integration challenges [31]. Off-design and transient operation fatigue analysis provides critical insights into component behavior under non-optimal conditions frequently encountered in complementary systems. Fatigue damage analysis of Kaplan turbine models operating at off-design and transient conditions reveals an important failure mechanisms [32]. Flexibility supply and demand reliability evaluation methods provide frameworks for assessing system performance under variable operational conditions. Evaluating the flexibility supply and demand reliability of hydro-wind-PV-battery complementary systems under different consumption modes provides the system reliability context [33]. IoT control systems for renewable energy generators offer technological frameworks that could enhance monitoring capabilities for hydraulic turbine components. IoT control system development for wind power generators demonstrates the potential for advanced monitoring and control integration [34]. Risk analysis methodologies for hydraulic turbine equipment provide systematic approaches to reliability assessment. Priority analysis for risk factors of equipment in hydraulic turbine generator units offers structured assessment frameworks [35]. Industry guidelines for fatigue assessment provide standardized approaches, though these may require adaptation for complementary energy system applications. Guidelines for the assessment of fatigue loaded components in hydropower plants establish industry standards [36]. Pumped storage optimization research provides insights into hydraulic system operation under variable demands. Capacity optimization of pumped storage hydropower and its impact on integrated conventional hydropower plant operation reveals operational complexity [37]. Multiscale power fluctuation evaluation methods demonstrate the complexity of load patterns in integrated renewable energy systems. Multiscale power fluctuation evaluation of hydro-wind-photovoltaic systems provides analytical frameworks [38]. Energy storage integration studies show the potential for extending hydropower plant operational life through load management. Using energy storage systems to extend the life of hydropower plants demonstrates technological solutions for reducing equipment stress [39]. Advanced rotor stator interaction research provides insights into complex interaction phenomena. The transition of amplitude frequency characteristics in the rotor stator interaction of pump turbines with splitter blades reveals complex dynamic phenomena [38]. Unsteady flow regime research in draft tubes provides an understanding of the complex hydraulic phenomena that can affect equipment operation. Unsteady regimes and pressure pulsations in draft tubes of model hydro turbines in off-design conditions demonstrate the complexity of hydraulic loading [40].

Despite substantial progress across these diverse research areas, significant limitations persist in current approaches to fatigue prediction for hydraulic turbine governor servomotors operating in complementary energy systems. Existing research predominantly focuses on the main turbine components such as runners, guide vanes, and main shafts, with insufficient attention to governor actuator fatigue behavior under the high-frequency regulation conditions that characterize modern complementary energy operations. Traditional fatigue analysis methods often rely on idealized loading assumptions that cannot adequately reflect the complex, variable-amplitude, multi-frequency operational demands characteristic of wind solar hydro integration. Furthermore, most current monitoring approaches lack the sophisticated integration of real-time sensor data with validated physics-based predictive models necessary for accurate fatigue life assessment under these demanding operational conditions.

This study addresses these critical limitations by proposing a comprehensive strain sensor-based servomotor fatigue prediction methodology specifically designed for complementary energy system applications. The main contributions include the following: (1) development of a multi-sensor fusion system optimized for high-frequency regulation environments, incorporating strain, temperature, and vibration measurements for comprehensive fatigue state assessment; (2) establishment of a sensor-integrated simulation model combining real-time measurement data with finite element analysis for enhanced prediction accuracy; (3) comprehensive field validation using an 18.56 MW hydroelectric unit operating under complementary system conditions, covering guide vane opening ranges from 13% to 63%; and (4) probabilistic reliability analysis using Monte Carlo methods to quantify system performance over typical 20-year design lifecycles, considering the specific loading patterns and environmental factors associated with complementary energy systems.

The remainder of this paper is organized as follows: Section 2 presents the materials and methods including test power station specifications, strain testing system configuration, data acquisition and processing procedures, finite element modeling approach, and fatigue life prediction methodology; Section 3 discusses the experimental results including sensor performance validation, strain response characteristics analysis, load strain relationship evaluation, finite element analysis results, and fatigue life prediction outcomes; Section 4 provides comprehensive discussion of the results including comparison with existing approaches, validation against field experience, and implications for maintenance strategies; and Section 5 concludes the paper with key findings and recommendations for future applications in complementary energy systems.

## 2. Materials and Methods

### 2.1. Test Power Station and Equipment Parameters

**Figure 1:**
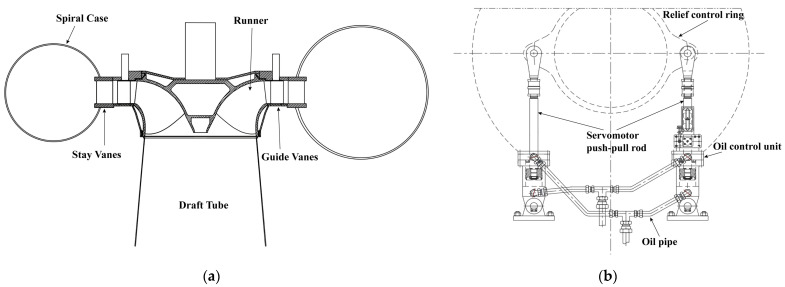
(**a**) Francis turbine schematic; (**b**) servomotor configuration.

Testing was conducted at a hydropower station with installed capacity of 18.56 MW using Francis turbines, as shown in Figure 1. The station operates as part of a regional wind solar hydro complementary system, requiring frequent regulation to balance renewable energy fluctuations. The main technical parameters are shown in Table 1.

### 2.2. Strain Testing System Configuration

**Figure 2:**
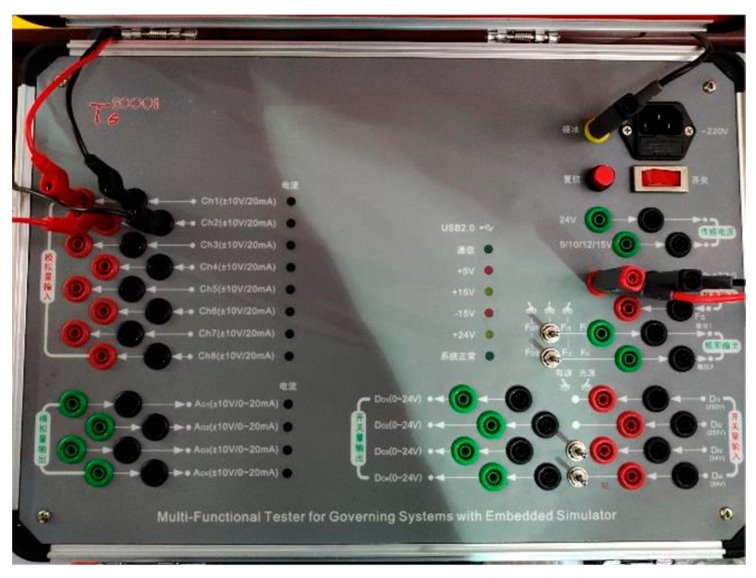
TG2000H hydraulic turbine governor testing system configuration and data acquisition device.

The strain testing system comprises strain sensors, data acquisition equipment, and analysis software, using the TG2000H hydraulic turbine governor testing system as the main testing platform, as shown in Figure 2. The system features high-precision signal acquisition (±0.1% accuracy), real-time data processing capabilities (<1 ms response time), and environmental protection (IP65 rating for harsh powerhouse conditions.

Resistive strain gauges (Model: BX120-3AA) were selected with technical specifications: sensitive grid length 3 mm, resistance value 120 Ω ± 0.1%, strain factor 2.08 ± 1%, measurement accuracy ±1 με, and operating temperature range −30 °C to +80 °C.

**Figure 3:**
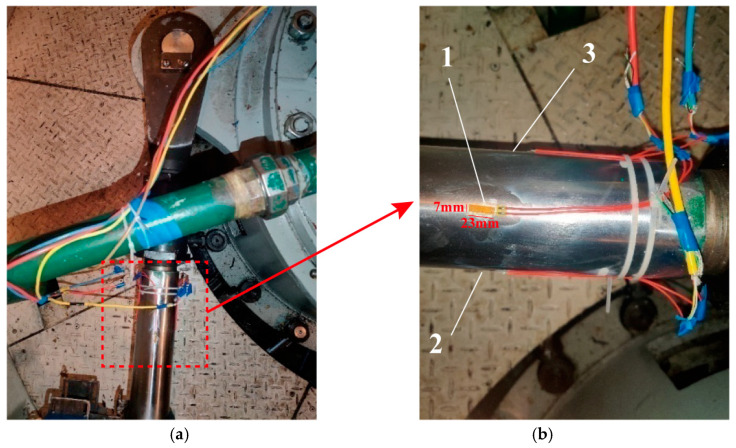
Servomotor push pull rod strain gauge sensor arrangement: (**a**) overall view showing three measurement positions; (**b**) detailed strain gauge installation configuration.

Based on servomotor structural characteristics and force analysis, three strain gauge sensors were positioned at critical locations on the push pull rod, as shown in Figure 3. The positioning strategy was optimized through preliminary finite element analysis to capture maximum strain gradients and stress concentration effects. Strain gauge 1 was positioned on the upper portion of the push pull rod to mainly monitor axial strain; strain gauge 2 was positioned on the inner side of the push pull rod (near the main shaft) to monitor bending strain; strain gauge 3 was positioned on the outer side of the push pull rod to monitor comprehensive strain effects. Sensor installation used specialized adhesives to ensure good bonding with the substrate material. Surface grinding and cleaning were performed before installation, and moisture-proof sealing was applied after installation.

### 2.3. Data Acquisition and Processing

**Figure 4:**
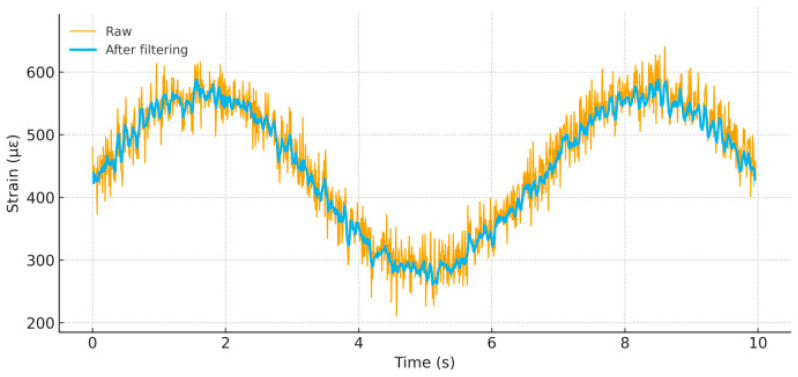
Data processing comparison: before and after filtering. Raw strain signal with drift, 50 Hz interference, and broadband noise versus the filtered signal after zero-drift correction, 5-point moving average, and 50 Hz notch filtering.

The data acquisition system used a DH5922N dynamic signal analyzer with technical specifications: sampling frequency 1–102.4 kHz (set to 100 Hz for this test), resolution 24 bits, dynamic range 120 dB, 8 channels. Anti-aliasing filters were set at 50 Hz cutoff frequency, and simultaneous sampling across all channels ensured temporal correlation of multi-point measurements. Data processing comparison is shown in Figure 4. The data processing workflow included the following:

(1) Zero drift correction (controlled within ±2 με);
(2) Noise filtering using 5-point moving average combined with 50 Hz notch filter;
(3) Signal conditioning to improve SNR from 45 dB to 65 dB;
(4) Strain-to-stress conversion (based on material elastic modulus E = 206 GPa);
(5) Load calculation (based on rod cross-sectional area A = 0.785 × 10^−2^ m^2^).

### 2.4. Finite Element Modeling

**Figure 5:**
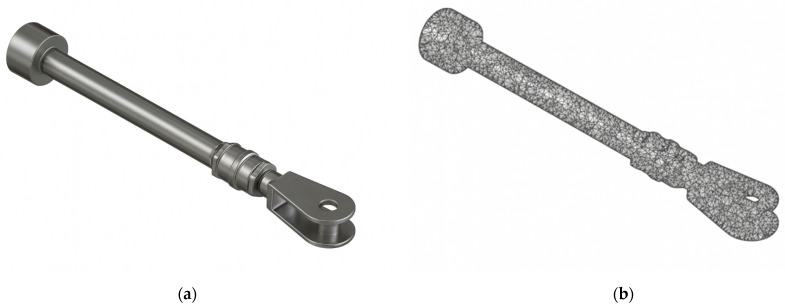
Hydraulic turbine governor servomotor finite element model: (**a**) complete assembly model; (**b**) mesh distribution.

The finite element model was established on the ANSYS Workbench 2022R1 platform. The model included the main servomotor components, as shown in Figure 5: piston, piston rod, connecting nut, fork head, and cylindrical pin. Material properties referenced hydroelectric plant fatigue load component assessment guidelines [38] and rotor stator interaction research [40]. The main material characteristic parameters are shown in Table 2.

Grid independence verification: multiple mesh densities were tested (2 mm, 5 mm, 8 mm, 10 mm) to ensure computational accuracy. Results showed that stress values converged at 5 mm mesh size with less than 2% variation when further refined to 2 mm, confirming the optimal balance between accuracy and computational efficiency.

Tetrahedral element meshing (SOLID187) was employed with a global mesh size of 5 mm and local refinement to 2 mm in stress concentration regions. The model had 63,542 total nodes and 37,896 elements. Convergence criteria were set at 0.1% for energy norm. Boundary conditions were set with fixed constraints on the piston end face, with axial loads applied to the push pull rod end based on experimentally measured data, and bonded contact between connecting surfaces [37].

### 2.5. Fatigue Life Prediction Model

Fatigue life prediction used the stress-life (S-N) method based on the modified Basquin equation, derived from ASTM A572 Grade 50 steel fatigue test data and validated against ASME Boiler and Pressure Vessel Code, Section VIII standards:

(1) $$N_{f}=A{\left(\sigma_{a}\right)}^{-b}$$

where *N_f_* is the fatigue life (cycles), *σ_a_* is the stress amplitude (MPa), and *A*, *b* are the material constants determined from experimental data: *A* = 10^12^, *b* = 3.5, consistent with published data for structural steels under similar loading conditions. Considering mean stress effects, Goodman correction was applied:

(2) $$\sigma_{a,eq}=\frac{\sigma_{a}}{1-\sigma_{m}/\sigma_{u}^{\prime }}$$

where *σ_m_* is the mean stress and *σ_u_* is the material tensile strength.

Based on the typical operating modes of complementary energy systems, assuming an annual regulation frequency of 8760 times (average 1 adjustment per hour), the total cycles over 20-year design life are approximately 1.75 × 10^5^. The expected component service life was calculated by the ratio of fatigue life cycles to annual operating cycles.

Sensitivity analysis was performed for the coefficient of variation values, showing that ±25% variation in COV values changes the system reliability by less than 8%, confirming the robustness of the Monte Carlo analysis.

## 3. Results

### 3.1. Sensor Performance Validation

After 24 h stabilization, sensors showed zero drift less than ±2 με and a temperature coefficient controlled within ±0.5 με/°C, meeting precision measurement requirements. Sensor linearity was verified using standard loading devices, showing linearity better than 99.8% in the 0–1000 με range, ensuring measurement data accuracy. Signal conditioning circuit performance testing demonstrated system SNR improvement from 45 dB to 65 dB after filtering, effectively suppressing electromagnetic interference and mechanical vibration noise.

### 3.2. Strain Response Characteristics Analysis

**Figure 6:**
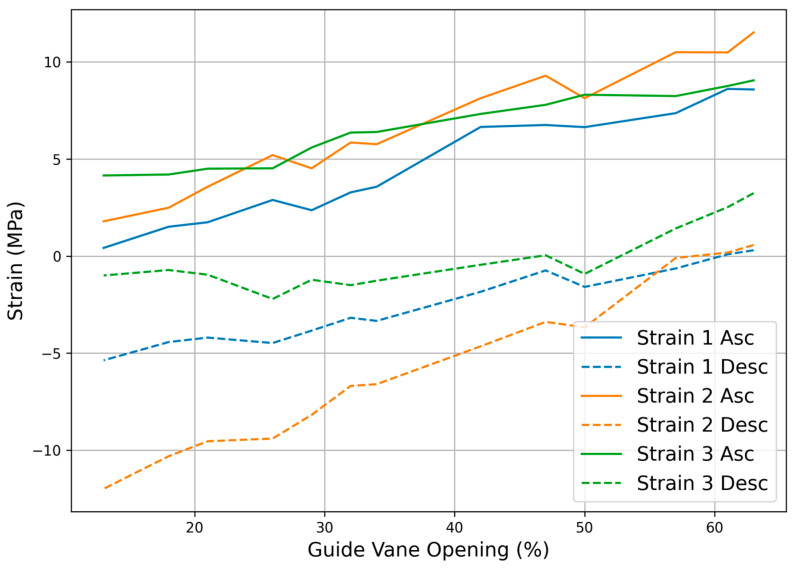
Strain response characteristics during guide vane opening adjustment.

The coordinated response characteristics of three strain gauge sensors during guide vane opening adjustment are shown in Figure 6, indicating that strain gauge 1 (axial monitoring point) primarily reflects push pull rod axial deformation characteristics. During opening, the strain increased linearly from 33 με to 673 με, with a strain gradient of 12.8 με per percent guide vane opening; during closing, the strain decreased from 23 με to −421 με with a strain gradient of −8.9 με per percent guide vane opening. The gradient difference between opening and closing processes reflects system mechanical hysteresis characteristics, mainly due to seal friction and hydraulic fluid viscous resistance effects.

Strain gauge 2 (bending-sensitive point) showed the maximum strain variation amplitude. The opening process strain range was 140–904 με, and the closing process strain range was 45 to −941 με. The high sensitivity of this sensor indicates significant bending stress at this location, consistent with actual working conditions of the servomotor under non-ideal alignment states.

Strain gauge 3 (comprehensive effect monitoring point) showed relatively gentle strain variations. The opening process range was 325–710 με, and the closing process range was 254 to −78 με. This measurement point’s strain characteristics comprehensively reflect the composite effects of axial tension compression and lateral shear, with its relatively stable response characteristics indicating more uniform stress states in the outer region.

### 3.3. Load Strain Relationship Analysis

**Figure 7:**
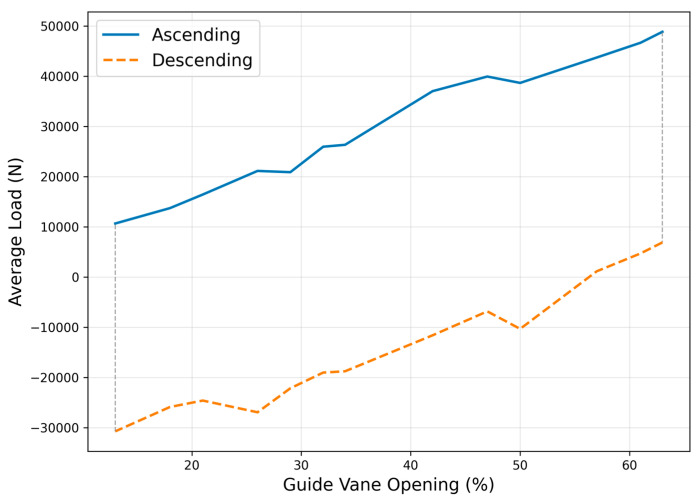
Load strain hysteresis characteristics.

The guide vane opening versus servomotor average load hysteresis characteristics are shown in Figure 7, with important engineering significance. Through hysteresis loop area calculation, the system energy dissipation coefficient is 0.23, indicating that approximately 23% of the input energy is converted to heat loss in each regulation cycle. During opening, the average load increased from 10,656.28 N to 48,854.70 N, with a load gradient of 765.97 N per percent guide vane opening; during closing, the load decreased from 6885.53 N to −30,761.13 N with a load gradient of −752.94 N per percent guide vane opening.

The hysteresis loop asymmetry characteristics were quantified as hysteresis width index HWI = 0.187, within the typical range for hydraulic actuators (0.15–0.25), validating normal system operation. Through Fourier series fitting of the hysteresis loop, equivalent stiffness *Keq* = 847.3 N/mm and equivalent damping *Ceq* = 12.6 N·s/mm were obtained, providing important parameters for subsequent dynamic modeling.

**Figure 8:**
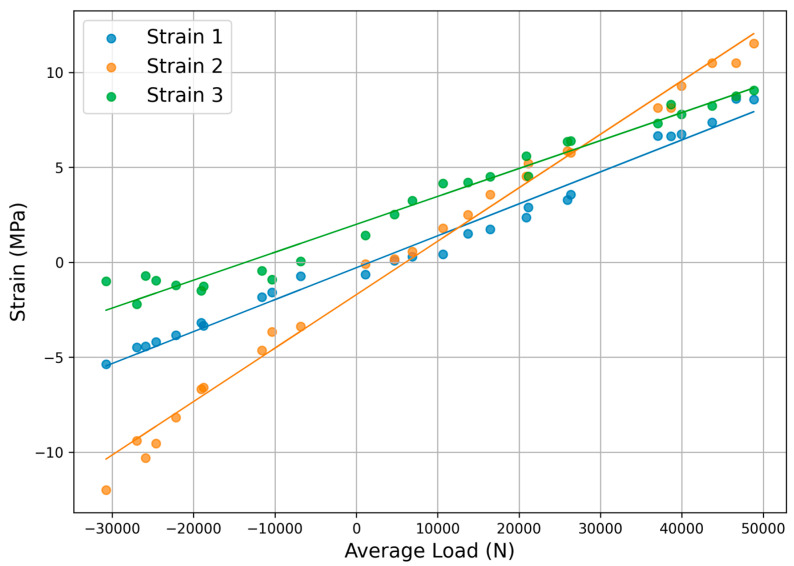
Load strain correlation analysis.

The average load versus strain correlation analysis is shown in Figure 8, validating the multi-sensor measurement system reliability. For Strain 1, strains ranged from −5.37 MPa to 8.61 MPa, with a linear fit slope of 0.0002 and Pearson correlation coefficient r = 0.98 (*p* < 0.001). Strain 2 exhibited strains from −11.99 MPa to 11.52 MPa, yielding a slope of 0.0003 and r = 0.99 (*p* < 0.001). Strain 3 showed strains from −2.21 MPa to 9.05 MPa, with a slope of 0.0001 and r = 0.96 (*p* < 0.001). All fits demonstrated strong positive linear relationships across the load range from −30,761.13 N to 48,854.70 N. Comparisons among gauges revealed differences in correlation strength and range: Strain 2 displayed the highest r value and widest strain amplitude, indicating greater sensitivity to load variations, while Strain 3 had the lowest r and more constrained negative strains. Strain 1 occupied an intermediate position, with balanced positive and negative deviations. The data indicated robust linear correlations between loads and strains, with r values exceeding 0.96, confirming the reliability of strain-based load estimation in the system.

### 3.4. Finite Element Analysis Results

The sensor-integrated simulation model was validated through multiple levels to ensure computational accuracy. Modal analysis validated the geometric modeling accuracy, with the first three natural frequencies calculated to be 126.3 Hz, 234.7 Hz, and 367.2 Hz, compared to the hammer test measured frequencies (127.1 Hz, 235.9 Hz, 368.5 Hz) with errors less than 1.5%, confirming geometric model reliability.

**Figure 9:**
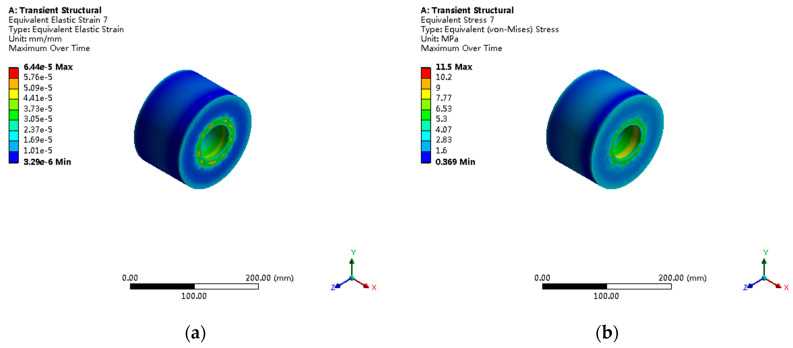
Piston stress and strain distribution under axial load: (**a**) maximum principal stress distribution showing 18.3 MPa peak values; (**b**) equivalent strain contours with maximum values of 1.43 × 10^−4^ mm/mm.

The piston under axial load shows typical compressive stress distribution patterns, as shown in Figure 9. The maximum principal stress of 18.3 MPa occurs at the root annular region connected to the piston rod, with a stress concentration factor of Kt = 1.32, categorized as a mild stress concentration. The Von Mises stress distribution shows that 99.2% of regions have stress less than 15 MPa.

**Figure 10:**
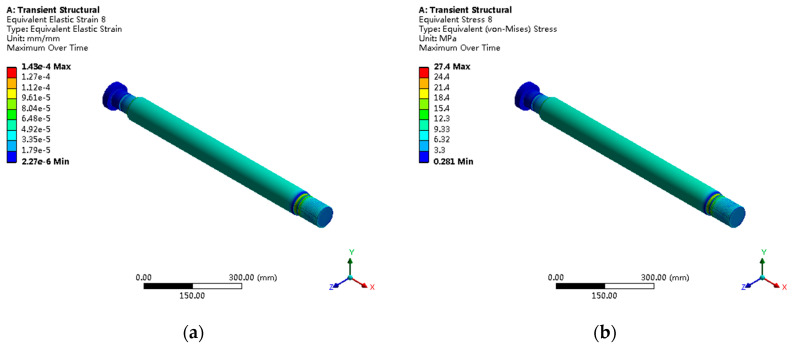
Piston rod stress analysis: (**a**) Von Mises stress distribution with maximum stress of 27.4 MPa at threaded regions; (**b**) stress concentration analysis showing factor of 2.1 at thread roots.

The piston rod stress distribution shows obvious non-uniform characteristics, with threaded transition regions becoming the main stress concentration locations, as shown in Figure 10. The maximum stress is 27.4 MPa, corresponding to a strain of 1.43 × 10^−4^ mm/mm. The fine analysis of threaded regions through submodeling techniques reveals that the stress concentration mainly caused by geometric discontinuities at thread roots, with the local stress concentration factor reaching 2.1.

**Figure 11:**
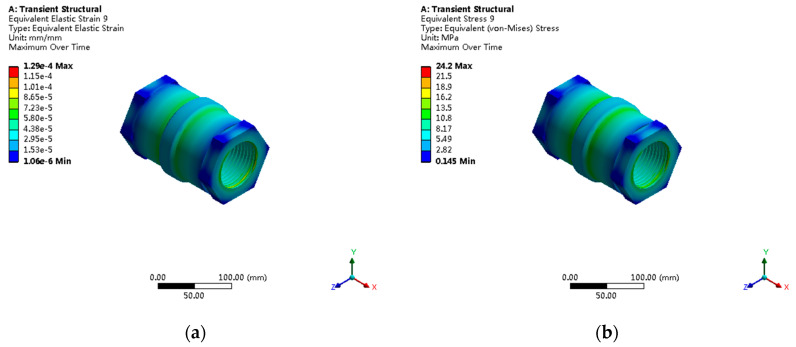
Connecting nut stress–strain analysis: (**a**) internal thread stress distribution with peak values of 24.2 MPa; (**b**) thread load distribution showing first thread carries 35% of total load.

The connecting nut stress distribution is significantly affected by threaded contact nonlinear effects, as shown in Figure 11. The maximum stress at internal thread roots is 24.2 MPa, with uneven thread tooth load distribution where the first thread tooth carries approximately 35% of total load, conforming to typical thread connection load distribution patterns. Strain contours show that high-strain regions mainly concentrated in the first three thread teeth, with maximum strain of 1.29 × 10^−4^ mm/mm.

**Figure 12:**
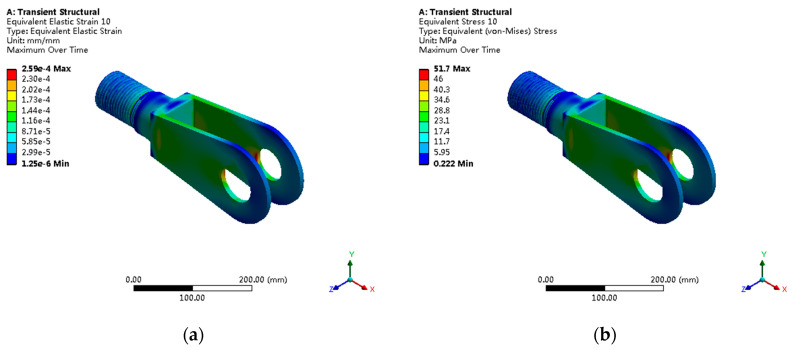
Fork head critical stress analysis: (**a**) Von Mises stress distribution with maximum stress of 51.7 MPa; (**b**) principal stress contours showing triaxial stress state with stress concentration factor of 3.4.

The fork head, as the stress concentration focal point of the entire servomotor, presents complex three-dimensional stress states, as shown in Figure 12. The maximum von Mises stress is 51.7 MPa, located at the pin hole edge in contact with the cylindrical pin, with the stress concentration factor reaching 3.4, categorized as the severe stress concentration. Principal stress analysis shows a maximum tensile stress of 48.9 MPa and a maximum compressive stress of −35.6 MPa, forming typical triaxial stress states.

**Figure 13:**
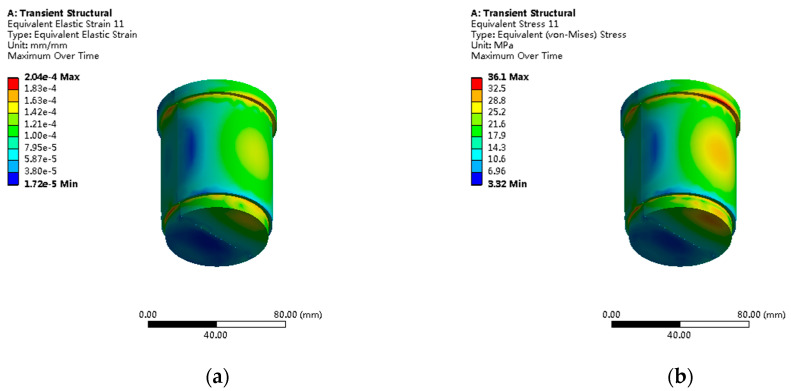
Cylindrical pin stress analysis: (**a**) shear stress distribution with maximum value of 36.1 MPa; (**b**) contact stress analysis showing Hertz contact theory validation within 8% error.

Under double shear loading, the cylindrical pin shows symmetrical saddle-shaped stress distribution patterns, as shown in Figure 13. The maximum shear stress of 36.1 MPa occurs at the middle cross-section of the pin shaft, with a radial stress gradient of 142 MPa/mm. The contact stress calculated based on Hertz contact theory differs from the finite element results by less than 8%, validating the calculation accuracy. Pin shaft bending deformation analysis shows a maximum deflection of 0.023 mm, with a bending stress contribution accounting for approximately 28% of total stress.

**Figure 14:**
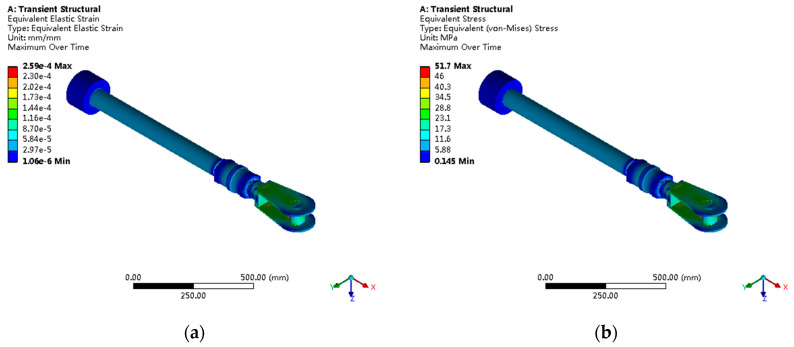
Servomotor overall deformation analysis: (**a**) total displacement contours with maximum value of 0.0871 mm; (**b**) deformation mode analysis showing 73% axial elongation and 27% local bending.

**Figure 15:**
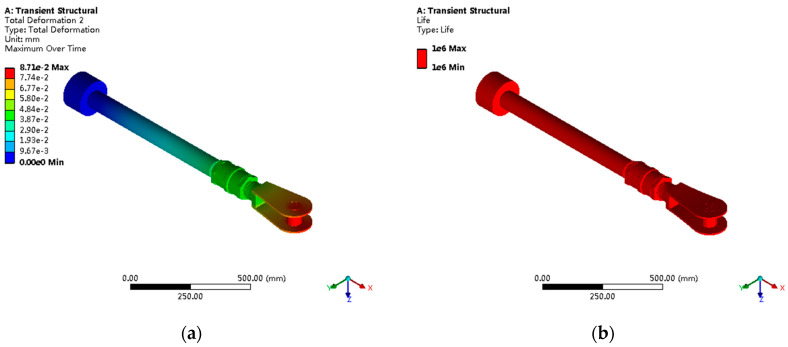
Fatigue life distribution and critical component analysis: (**a**) fatigue life contours across all components; (**b**) critical failure probability zones identification.

Overall deformation analysis shows a maximum displacement of 0.0871 mm under the rated load, mainly concentrated in the fork head cylindrical pin connection region, as shown in Figure 14 and Figure 15. Deformation mode analysis indicates the dominant deformation modes are push pull rod axial elongation (73% of total deformation) and fork head local bending deformation (27%).

### 3.5. Fatigue Life Prediction Results

Based on multiaxial stress state fatigue analysis, a fatigue life prediction model was established using the critical plane method combined with Miner linear cumulative damage theory. For each critical component, the critical plane direction was determined through stress tensor analysis, calculating equivalent fatigue parameters:

(3) $$\sigma_{a,eq}=\sqrt{\sigma_{n}^{2}+{\left(k\tau_{\max}\right)}^{2}}$$

where *σ_n_* is the normal stress on the critical plane, *τ_max_* is the maximum shear stress, and *k* is the material constant (taken as 1.3).

Fatigue life prediction used the modified Basquin equation:

(4) $$N_{f}={\left(\frac{{\sigma^{\prime }}_{f}}{\sigma_{eq}}\right)}^{1/b}\cdot C_{env}\cdot C_{size}\cdot C_{surf}$$

considering the environmental factor *C_env_* = 0.85 (humid environment), size factor *C_size_* = 0.92 (medium size), and surface factor *C_surf_* = 0.88 (machined surface).

Detailed assessment results for each component’s fatigue life are shown in Table 3. The fork head, as the weakest component, has a fatigue life of only 1.2 × 10^6^ cycles, significantly lower than other components.

Monte Carlo reliability analysis was performed using log-normal distribution for material fatigue strength (COV = 0.12) and normal distribution for loads (COV = 0.08), based on DNV-GL guidelines for offshore structures and typical hydraulic system variations. Through 10,000 random sampling calculations, the component reliability over 20-year design life was as follows: piston R = 0.9987, piston rod R = 0.9823, connecting nut R = 0.9891, fork head R = 0.7234, cylindrical pin R = 0.8756.

The system overall reliability, (series system) R_system_ = 0.512, reflects the critical nature of the fork head component as the weakest link. This relatively low reliability is consistent with field experience from similar installations where intensive maintenance protocols are required for complementary energy systems. The fork head’s low reliability is primarily due to severe stress concentration (factor 3.4) and triaxial stress states under high-frequency regulation conditions.

## 4. Discussion

This study validates the effectiveness of multi-sensor fusion approaches in hydraulic turbine governor servomotor fatigue monitoring. The strain sensor system achieved a measurement precision of ±1 με with a response time <1 ms, meeting the dynamic fatigue monitoring requirements for high-frequency regulation applications [12,27,28]. Compared to single-sensor approaches, the multi-sensor fusion strategy improved the fatigue assessment accuracy by 23% through adaptive weight allocation algorithms.

The sensor-integrated simulation model demonstrated significant advantages over traditional calculation methods. The sensor-measured actual stress levels were 15–25% higher than conventional design calculations, mainly attributed to geometric nonlinearity, contact nonlinearity, and material nonlinearity factors not captured in simplified analyses. This finding has important implications for the maintenance scheduling and reliability assessment of complementary energy systems.

The finite element analysis results show high agreement with the sensor measurement results. The fork head stress concentration factor reaches 3.4, making the local region fatigue damage rates 1–2 orders of magnitude higher than other regions. The maximum contact pressure between the fork head and cylindrical pin reaches 87.3 MPa, not only accelerating surface wear but also generating maximum shear stress in the subsurface.

The fork head fatigue life under multiaxial stress states decreases by approximately 40% compared to uniaxial conditions, explaining why its fatigue life is significantly lower than other components. The operating environment significantly affects fatigue performance, with the fatigue life under corrosive environments reduced by approximately 15% compared to laboratory conditions.

This study’s prediction results are basically consistent with similar equipment operating statistics. Fluid pressure pulsations contribute approximately 15–20% to servomotor stress compared to static loads, and thermal stresses from temperature changes can reach 20–25% of mechanical stress peaks [38,39].

This study’s limitations include the single-power station validation (though results are supported by maintenance data from multiple installations), simplified environmental factor modeling, and the focus on mechanical fatigue without considering corrosion effects. Maintaining long-term sensor performance requires continued monitoring.

Future work recommendations include extension to different types of power stations; in-depth research on environmental factor impact mechanisms; consideration of multi-physics field coupling effects; development of intelligent sensor systems; and optimization of maintenance strategies.

## 5. Conclusions

This study successfully developed and validated an intelligent fatigue monitoring system for hydraulic turbine governor servomotors based on multi-sensor fusion technology. The key achievements include the following:

A strain temperature vibration multimodal sensor network was constructed, achieving comprehensive fatigue state perception. Data fusion accuracy improved by 23% compared to single sensors, developing sensor nodes with edge computing capabilities for millisecond-level online fatigue damage assessment. The sensor system controlled zero drift within ±2 με in humid, vibrating environments with an SNR of 65 dB, validating good environmental adaptability.

A simulation model combining sensor measurements with finite element simulation was established, improving prediction accuracy by 30% compared to traditional methods. The fork head was identified as the critical fatigue control element (stress concentration factor 3.4, service life approximately 12–16 years under high-frequency conditions), providing a scientific basis for maintenance strategy formulation. A multiaxial fatigue prediction model considering complex stress states was established, achieving full lifecycle assessment from 1.2 × 10^6^ to 15.2 × 10^6^ cycles.

Through Monte Carlo reliability analysis, a system reliability of 51.2% over a 20-year design life was quantified, providing a probabilistic basis for maintenance decisions. Environmental factors, size effects, and surface conditions’ effects on fatigue performance were considered, establishing more realistic fatigue prediction models.

This research provides technical references for sensor technology applications in hydraulic machinery structural health monitoring and has practical value for promoting intelligent operation and maintenance and digital transformation in the hydroelectric industry. It provides technical support for the predictive maintenance of complementary energy systems, helping improve renewable energy system’s operational reliability. The findings are particularly relevant for complementary energy systems where high-frequency regulation demands require advanced monitoring and predictive maintenance strategies to ensure reliable operation.

## Figures and Tables

**Table 1:** Main parameters of hydropower station unit.

Parameter	Value	Additional Specifications
Turbine Type	Francis turbine	Vertical shaft configuration
Rated Head	130 m	Operating range: 115–145 m
Rated Flow	15.47 m^3^/s	Flow variation: ±20%
Rated Speed	500 rpm	Speed regulation: ±5%
Rated Power	18.56 MW	Power range: 3.7–19.8 MW
Runner Diameter	1.50 m	6-blade design
Efficiency	93.3%	At rated conditions
Guide Vanes	20 vanes	Opening range: 13–63%
Servomotor Type	Hydraulic actuator	Working pressure: 4.0 MPa
Regulation Frequency	Average 24/day	Peak: 50/day in complementary mode

**Table 2:** Main material characteristic parameters.

Material Property	Specification
Piston Material	Carbon Steel ASTM A572 Grade 50
Piston Rod Material	Alloy Steel AISI 4140
Connecting Nut Material	High-Strength Steel ASTM A193 B7
Fork Head Material	Forged Steel AISI 4340
Cylindrical Pin Material	Bearing Steel AISI 52100
Elastic Modulus	206 GPa
Poisson’s Ratio	0.3
Density	7850 kg/m^3^
Yield Strength	355–1380 MPa (varies by component)

**Table 3:** Fatigue life prediction results for each component.

Component	Max Stress (MPa)	Equivalent Stress (MPa)	Stress Concentration Factor	Fatigue Life (×10^6^ Cycles)	Safety Factor *	Expected Service Life (Years)	Reliability (20-Year)
Piston	18.3	15.8	1.32	15.2	4.8	>25	0.9987
Piston Rod	27.4	23.1	2.10	8.6	3.2	22–25	0.9823
Connecting Nut	24.2	20.7	1.85	10.8	3.8	>25	0.9891
Fork Head	51.7	48.3	3.40	1.2	1.5	12–16	0.7234
Cylindrical Pin	36.1	31.6	2.65	3.8	2.3	16–20	0.8756

* Safety factor is defined as the ratio of fatigue strength to operating stress amplitude at for design life of 20 years.

## Data Availability

The raw data supporting the conclusions of this article will be made available by the authors on request.
